# CRISPR/Cas9 offers a new tool for studying the role of chromatin architecture in disease pathogenesis

**DOI:** 10.1186/s13059-018-1569-z

**Published:** 2018-11-06

**Authors:** Xiang Guo, Ann Dean

**Affiliations:** Laboratory of Cellular and Developmental Biology, National Institute of Diabetes and Digestive and Kidney Diseases, National Institutes of Health, Bethesda, MD 20892 USA

## Abstract

A recent study used CRISPR/Cas9 to reveal long-range looping between disease-related genes and their regulatory elements that is mediated by the CCCTC-binding factor (CTCF) in prostate cancer.

## Introduction

Extensive studies are currently devoted to understanding the three-dimensional (3D) architecture of genomes, including the formation and function of chromatin loops, topologically associated domains (TADs) and transcriptional activity-based A and B compartments. These studies provide evidence that the spatial organization of the genome is a global regulator of gene transcription. DNA looping is the fundamental architectural unit of the 3D genome and builds long-range connections and communication between genes and their regulatory enhancer elements. In addition, genome-wide association studies (GWAS) have revealed that a large fraction of disease-associated mutations or genomic rearrangements are found in non-coding rather than coding regions of the genome, providing a clue that the long range communication between genes and regulatory elements might play an important role in human disease [1]. A recent article presents a systemic approach for connecting these genomic aberrations to underlying disease genes in order to understand how the GWAS-identified single nucleotide polymorphisms (SNPs) are related to disease [2].

## Chromosomal looping in prostate cancer

Enhancer-promoter looping facilitates the assembly of the transcriptional machinery at specific promoters and thus the effective initiation of transcription. Histone H3K27ac distinguishes active enhancers, which are the ones that frequently engage in looping to transcriptionally active genes, from inactive or poised enhancers. Chromosomal looping is also maintained by numerous architectural proteins and by long non-coding RNAs (lncRNAs) in addition to regulatory DNA elements. The major architectural protein CCCTC-binding factor (CTCF) is an 11 zinc-finger DNA-binding protein that associates with the cohesin complex and orchestrates long range interactions between remote enhancers and their target gene promoters to modulate gene transcription. Whether this regulation is direct or indirect and involving an insulator function is an open question.

In this issue of *Genome Biology*, Guo et al. [2] describe how prostate cancer (PCa) risk loci that they identified in GWAS participate in CTCF-mediated chromatin loops and function to repress the expression of the encircled genes. Strikingly, deletion of the PCa-associated CTCF loop anchors using the CRISPR/Cas9 (clustered regularly interspersed palindromic repeats/CRISPR-associated system 9) genome editing approach resulted in up to 100-fold increases in the expression of some genes within the CTCF loops. In one case, the upregulated gene, *KCNN3* (also called *SK3*), was already known to be linked to PCa biology. The CRISPR editing results suggest that *KCNN3* is normally held in a repressive loop by CTCF, revealing a novel mechanism underlying genetic susceptibility to PCa.

Guo and colleagues set out to discover regulatory elements that are associated with 2181 PCa risk-associated SNPs [2]. First, they required that the PCa-risk SNPs reside in open chromatin, as determined by DNase I hypersensitivity, which reduced the number of potential regulatory-function-associated SNPs to 443. They then compared these 443 SNPs to the ChIP-seq peaks for the active enhancer marker H3K27ac or insulator protein CTCF in two non-tumorigenic prostate cell populations and five prostate cancer cell lines. In this way, they identified 222 PCa-risk-associated SNPs corresponding to an H3K27ac peak and 93 corresponding to a CTCF peak, suggesting an enhancer- or insulator-like function of these SNPs in PCa.

To ask whether the enhancer- or insulator-related SNPs were involved in the regulation of chromosomal architecture, Guo et al. [2] employed in situ Hi-C to detect long-range looping interactions in normal prostate RWPE-1 cells and in the PCa cell lines C4-2B and 22Rv1. After overlap with loop anchor regions, 203 H3K27ac-associated SNPs and 85 CTCF-associated SNPs were identified as participants in chromatin loops. To delve more deeply into the functional relationships among regulatory elements, chromosome folding and the transcription landscape, the authors deleted CTCF loop anchor regions that contained PCa-risk SNPs on chromosomes 1 and 12 by CRISPR/Cas9 and performed transcriptome analysis before and after deletion. On chromosome 1, loop-encircled *KCNN3* expression increased almost 100-fold while other nearby genes were unaffected. On chromosome 12, *KRT78* was activated more than 100-fold, while the expression of some neighboring genes was modestly elevated. In contrast to these deletions, when the CTCF sites that paired with the PCa risk-associated CTCF anchor regions on the two chromosomes were deleted, there was only a modest or no influence on gene expression within the loop.

**Fig. 1:**
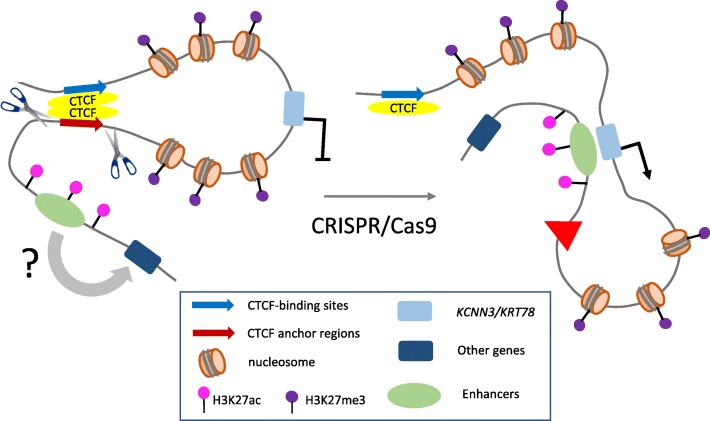
Model illustrating the chromatin architectural basis for the aberrant activation of gene expression in prostate cancer. Regions that are associated with prostate cancer risk bind CTCF and interact with each other to maintain the repression of genes within a looped region. Chromatin surrounding the gene is marked by repressive histone H3K27me3. When a prostate cancer risk-associated CTCF anchor region (red arrow becomes red triangle) is deleted by CRISPR/Cas9-based editing, the putative CTCF-mediated loop is no longer formed, and a formerly repressed gene can be accessed and aberrantly activated by an enhancer, marked by H3K27ac, that is located outside the former loop

What underlies the gene activation? Both *KCNN3* and *KRT78* are in genomic regions that are devoid of the H3K27ac mark. The authors investigated whether an enhancer element within the looped regions had become activated as assessed by H3K27ac acquisition but did not detect such a change. Instead, the result suggests that after the deletion, the genes came under the influence of a pre-existing enhancer outside the CTCF-mediated loop area that was compatible with only some of the genes that were newly available to it. As Guo et al. [2] propose, loss of an ‘insulator’ loop that had blocked the excluded enhancer, and the establishment of interaction between the activated genes and such an enhancer, could explain this result nicely (Fig. 1). Hi-C was not carried out for the deleted cells, so this idea remains speculative. Structural studies after the deletions could provide support for this idea and might also reveal what new CTCF–CTCF associations occur that could help to explain why the deletion of one loop anchor participant was more effective than that of another in boosting *KCNN3* or *KRT78* transcription.

## Putting CRISPR to work in the 3D genome

The 100-fold activation of *KCNN3* and *KRT78* after deletion of the two PCa-risk-associated CTCF anchor regions is especially notable in contrast to results obtained upon rapid removal of CTCF in embryonic stem cells [3]. In this work, the effects on the transcriptome were generally modest and only a few genes were upregulated more than ten-fold. On the other hand, both groups concluded that those genes that are upregulated upon CTCF loss are normally repressed by CTCF indirectly. One caveat to this definitive conclusion is that, considering the size of the CTCF anchor region deletions (1–2 kb) in the current work, a role for other factors bound along with CTCF cannot be excluded. To address the true function of CTCF in gene regulation, more precise CRISPR/Cas9 editing may be required.

CRISPR/Cas9 technology is proving useful in creating precise deletions or mutations of CTCF motifs in order to determine CTCF function in cell-fate determination, gene regulation and genome topology. A nine-base pair (bp) homozygous deletion was generated by CRISPR/Cas9 editing in a core CTCF motif at a boundary in the *HoxA* cluster that functions to separate adjacent TADs. Abrogation of CTCF occupancy at this position resulted in the *Hox* genes’ becoming subject to transcriptional activation from outside their original domain [4]. Consistent with Guo et al. [2], CTCF functions as a regulator by long-range looping to insulate the *Hox* cluster’s repressive genomic region from active chromatin and thus to maintain a low level of gene expression. In addition, a recent study showed that CRISPR-mediated disruption of CTCF-binding sites at an α-globin locus, subTAD, allowed the α-globin enhancers to activate genes on the other side of the lost boundary, which they normally cannot access [5]. Another study developed a CRISPR/Cas9-based DNA-fragment in-situ-inversion technology and demonstrated that the relative orientation of CTCF-binding sites in enhancers and promoters determined the directionality of DNA looping and the regulation of gene expression [6].

CRISPR/Cas9 has been more broadly employed to determine how 3D genome architecture is related to disease states. For example, CRISPR/Cas9 deletion of CTCF sites at the boundaries of an insulated neighborhood containing proto-oncogenes was sufficient to induce their activation in non-malignant cells [7]. Importantly, recurrent focal deletions in the same regions are associated with the expression of oncogenes in T-cell acute lymphoblastic leukemia. Disruption of a CTCF-binding motif between TADs by CRISPR/Cas9 resulted in the loss of insulation and in aberrant enhancer activation of a receptor tyrosine kinase gene, *PDGFRA*, leading to an enhanced gliomagenesis [8]. In another example, investigators used CRISPR to delete a CTCF boundary element, thereby creating topological changes, and were able to recreate a known human genetic limb malformation in a mouse model [9]. A recent innovative extension of the CRISPR approach, the CRISPR-dCAS9 CLOuD9 system, which involves plant phytohormone S-(+)-abscisic acid (ABA)-induced proximity reagents to reconfigure loops between enhancers and promoters, should provide a widely applicable way to re-engineer genome conformation [10].

Defining genome architectural mechanisms in disease-related gene regulation has great potential to illuminate the role of GWAS-identified noncoding variation in cis-regulatory elements and regulators of DNA topology. On the basis of the systematic approach described by Guo et al. [2], CRISPR/Cas9 editing technology will be highly valuable in the creation of additional disease models and is likely to provide new insight into 3D architectural-based gene therapy.

## Abbreviations

Cas9: CRISPR-associated system 9
CRISPR: Clustered regularly interspersed palindromic repeats
CTCF: CCCTC-binding factor
GWAS: Genome-wide association studies
PCa: Prostate cancer
SNP: Single nucleotide polymorphism
TAD: Topologically associated domain
